# Endothelial Ion Channels and Cell-Cell Communication in the Microcirculation

**DOI:** 10.3389/fphys.2022.805149

**Published:** 2022-02-08

**Authors:** William F. Jackson

**Affiliations:** Department of Pharmacology and Toxicology, College of Osteopathic Medicine, Michigan State University, East Lansing, MI, United States

**Keywords:** endothelial ion channels and cell-cell communication ion channels, endothelial cells, arterioles, vascular smooth muscle cells, pericytes, capillaries, blood flow, functional hyperemia

## Abstract

Endothelial cells in resistance arteries, arterioles, and capillaries express a diverse array of ion channels that contribute to Cell-Cell communication in the microcirculation. Endothelial cells are tightly electrically coupled to their neighboring endothelial cells by gap junctions allowing ion channel-induced changes in membrane potential to be conducted for considerable distances along the endothelial cell tube that lines arterioles and forms capillaries. In addition, endothelial cells may be electrically coupled to overlying smooth muscle cells in arterioles and to pericytes in capillaries *via* heterocellular gap junctions allowing electrical signals generated by endothelial cell ion channels to be transmitted to overlying mural cells to affect smooth muscle or pericyte contractile activity. Arteriolar endothelial cells express inositol 1,4,5 trisphosphate receptors (IP_3_Rs) and transient receptor vanilloid family member 4 (TRPV4) channels that contribute to agonist-induced endothelial Ca^2+^ signals. These Ca^2+^ signals then activate intermediate and small conductance Ca^2+^-activated K^+^ (IK_Ca_ and SK_Ca_) channels causing vasodilator-induced endothelial hyperpolarization. This hyperpolarization can be conducted along the endothelium *via* homocellular gap junctions and transmitted to overlying smooth muscle cells through heterocellular gap junctions to control the activity of voltage-gated Ca^2+^ channels and smooth muscle or pericyte contraction. The IK_Ca_- and SK_Ca_-induced hyperpolarization may be amplified by activation of inward rectifier K^+^ (K_IR_) channels. Endothelial cell IP_3_R- and TRPV4-mediated Ca^2+^ signals also control the production of endothelial cell vasodilator autacoids, such as NO, PGI_2_, and epoxides of arachidonic acid contributing to control of overlying vascular smooth muscle contractile activity. Cerebral capillary endothelial cells lack IK_Ca_ and SK_Ca_ but express K_IR_ channels, IP_3_R, TRPV4, and other Ca^2+^ permeable channels allowing capillary-to-arteriole signaling *via* hyperpolarization and Ca^2+^. This allows parenchymal cell signals to be detected in capillaries and signaled to upstream arterioles to control blood flow to capillaries by active parenchymal cells. Thus, endothelial cell ion channels importantly participate in several forms of Cell-Cell communication in the microcirculation that contribute to microcirculatory function and homeostasis.

## Introduction

The microcirculation is the business-end of the cardiovascular system. It is here that oxygen, substrates, hormones, etc. are supplied to the parenchymal cells to meet their metabolic and other physiological demands (Durán et al., 2011). In most tissues and organs, blood flow to the microcirculation is regulated to exactly match the metabolic demands of the perfused parenchyma such that there is a linear relationship between tissue oxygen consumption (metabolic demand) and tissue blood flow (supply of oxygen and other substrates; Laughlin et al., 2011). This tight coupling between blood flow and tissue metabolism strongly depends on cell-cell communication among the cells that comprise the blood vessels that make up the microcirculation as well as the surrounding parenchymal cells. This perspective will focus on the function of resistance artery, arteriolar, and capillary endothelial cell ion channels and how they contribute to cell-cell communication involved in control of blood flow in the microcirculation.

Endothelial cells express a diverse array of ion channels that importantly contribute to their function and especially cell-cell communication (Jackson, 2016; Garcia and Longden, 2020). These vascular cells are tightly coupled by homocellular (EC-EC) gap junctions such that they function as an electrical syncytium allowing changes in endothelial cell membrane potential to be conducted long distances (millimeters) along capillaries and arterioles in the microcirculation (de Wit and Griffith, 2010; Socha et al., 2012a). Endothelial cells also are electrically coupled to overlying smooth muscle cells (in arterioles and resistance arteries) or pericytes (in capillaries) by heterocellular gap junctions, modulating the membrane potential and contraction of these mural cells to control microvascular blood flow (de Wit and Griffith, 2010; Socha et al., 2012a; Molica et al., 2018). Ion channels in endothelial cell plasma and smooth endoplasmic reticulum membranes are responsible for generating ionic currents that either produce or modulate membrane potential, an important signal for cell-cell communication in the microcirculation (de Wit and Griffith, 2010; Socha et al., 2012a). In addition, membrane potential affects the electrochemical gradient for diffusion of ions through all ion channels (Nilius and Droogmans, 2001). Thus, membrane hyperpolarization, for example, will augment Ca^2+^ influx through Ca^2+^ permeable channels, whereas membrane depolarization will have the opposite effect.

Intracellular Ca^2+^ is another signal for cell-cell communication that is generated by ion channels (de Wit and Griffith, 2010; Edwards et al., 2010; Socha et al., 2012a; Jackson, 2016). Calcium signals can be transmitted through gap junctions to neighboring cells (de Wit and Griffith, 2010; Socha et al., 2012a,b) and, in endothelial cells, intracellular Ca^2+^ controls the production of endothelial cell autacoids (NO, PGI_2_, epoxides of arachidonic acid, H_2_O_2_, etc.) that themselves are important signals for cell-cell communication in the wall of microvessels (Edwards et al., 2010; Socha et al., 2012a; Jackson, 2016). Thus, endothelial cell ion channels play a central role in cell-cell communication and regulation of blood flow in the microcirculation.

**Figure 1:**
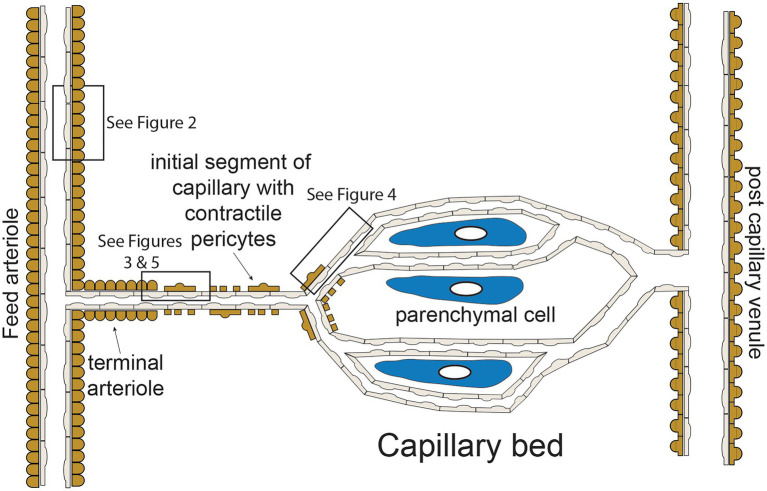
Microvascular unit—schematic representation of hypothetical microvascular unit that consists of a feed arteriole that branches into a terminal arteriole. The terminal arteriole transitions into an initial capillary segment that is coated with contractile pericytes including at the initial branch points, as shown. As the capillaries progress, they lose coverage by contractile pericytes which are replaces by non-contractile pericytes (not shown in the figure for clarity). As shown, the capillaries are in close association with parenchymal cells. The capillaries then converge and eventually drain into venules, which are coated in pericytic smooth muscle cells that are morphologically distinct from arteriolar smooth muscle cells. Boxes in the figure depict regions corresponding to Figures 2–5 as shown. See text for details and references.

**Figure 2:**
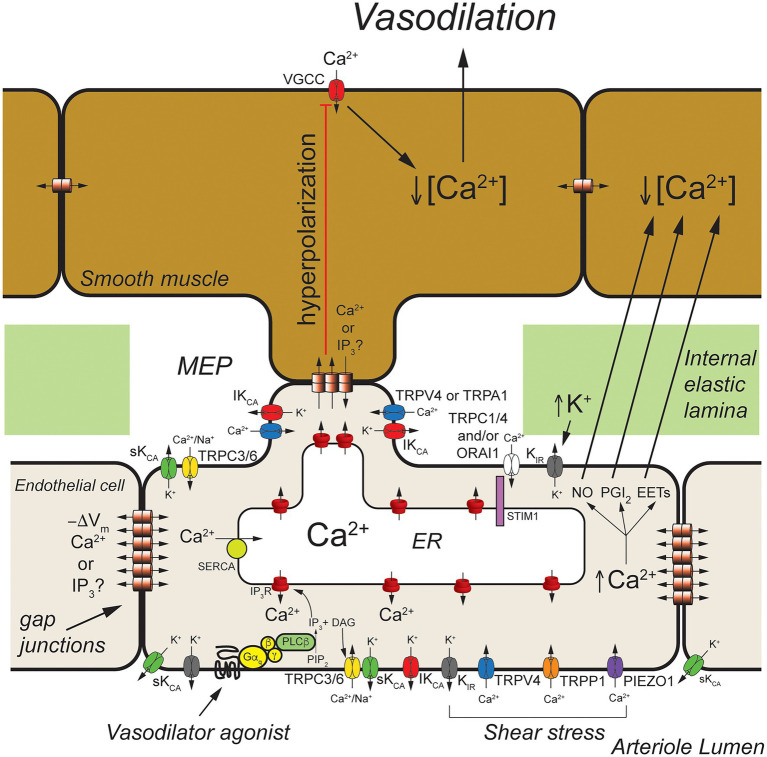
Arteriolar endothelial ion channels and cell-cell communication. Shown is a schematic representation of a longitudinal cross-section through one wall of an arteriole showing cross-sections of endothelial cells and overlying smooth muscle cells. Endothelial cells communicate with overlying smooth muscle cells at myoendothelial projections (MEPs) that pass through the internal elastic lamina to make contact with overlying smooth muscle cells, as shown. Gap junctions may form at MEPs to yield myoendothelial gap junctions (MEGJs) allowing endothelial cell hyperpolarization to be conducted to the smooth muscle cells, closing smooth muscle voltage-gated Ca^2+^ channels (VGCCs), decreasing [Ca^2+^]_in_, and leading to vasodilation. At MEPs transient receptor potential vanilloid family member 4 (TRPV4), intermediate conductance Ca^2+^-activated K^+^ (IK_Ca_) channels and inositol 1,4,5 trisphosphate receptors (IP_3_R) are clustered forming signaling complexes to direct the endothelial cell responses to vasodilator agonists. In cerebral arteries/arterioles, transient receptor potential ankyrin family member 1 (TRPA1) is also in these complexes. Other ion channels, such as transient receptor potential C family member 3 (TRPC3) channels and small conductance Ca^2+^-activated K^+^ (SK_Ca_) channels, may cluster elsewhere to form other signaling complexes. Endothelium-dependent vasodilators, such as acetyl choline, act on Gα_q_-coupled receptors to activate phospholipase C-β (PLCβ) which hydrolyses membrane phosphatidylinositol 4,5-bisphosphate (PIP_2_) forming IP_3_ and diacylglycerol (DAG). The IP_3_ activates IP_3_Rs to release Ca^2+^ from the ER increasing [Ca^2+^]_in_. The DAG activates membrane TRPC3 and TRPC6 channels which serve as receptor-operate channels conducting Na^+^ and Ca^2+^ into the cells. DAG also activates protein kinase C (PKC; not shown) which phosphorylates and activates TRPV4 channels. Calcium influx through TRPV4 channels acts on IP_3_-sensitized IP_3_R, amplifying Ca^2+^ influx through the TRPV4 channels. At MEP, the increase [Ca^2+^]_in_ results in activation of IK_Ca_ channels causing membrane hyperpolarization (−ΔV_m_) that is transmitted through MEGJs to hyperpolarize overlying smooth muscle causing vasodilation. Transient receptor potential C family members 1 and 4 (TRPC1 and TRPC4), and ORAI1 channels are activated upon release of Ca^2+^ from the endoplasmic reticulum (ER) *via* IP_3_Rs that is sensed by stromal interaction molecule 1 (STIM1) in the ER membrane. Inward rectifier K^+^ (K_IR_) channels (likely K_IR_2.1) are expressed and can be activated by membrane hyperpolarization to facilitate hyperpolarization induced by SK_Ca_ and IK_Ca_ channels. They also are activated by increases in extracellular K^+^ and involved with sensing shear stress. Shear stress also appears to activate TRPV4 channels, transient receptor potential polycystin family member 1 (TRPP1) channels, and PIEZO1 channels leading to increased endothelial [Ca^2+^]_in_ and vasodilation. In addition to activating SK_Ca_ and IK_Ca_ channels to produce hyperpolarization-induced vasodilation, increased endothelial cell [Ca^2+^]_in_ also results in production of endothelial cell vasodilator autacoids, such as nitric oxide (NO), prostacyclin (PGI_2_), and epoxides of arachidonic acid (EETs) that cause arteriolar vasodilation. See text for additional information and references.

**Figure 3:**
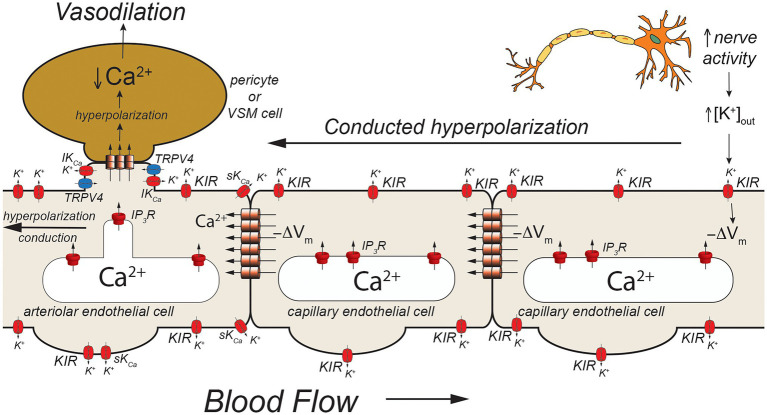
Capillaries as sensors of [K^+^]_out_. Schematic of a capillary segment that transitions to a vessel invested with contractile pericytes (initial capillary segment) or smooth muscle cells (terminal arteriole). Active neurons release K^+^ during action potential repolarization. This increases [K^+^]_out_ which is sensed by K_IR_2.1 channels (KIR). Movement of K^+^ out of activated K_IR_2.1 channels results in membrane hyperpolarization (−ΔV_m_). The local membrane hyperpolarization activates adjacent K_IR_2.1 channels allowing this hyperpolarization to be conducted from the site of initiation through homocellular gap junctions from endothelial cell-to-endothelial cell back toward the initial capillary segment (invested with contractile pericytes) and the terminal arteriole (with smooth muscle cells). These contractile mural cells are coupled to underlying endothelial cells by heterocellular gap junctions which allow transmission of the hyperpolarization to the contractile cells. In smooth muscle cells (and presumably contractile pericytes), the hyperpolarization will deactivate voltage-gated Ca^2+^ channels, decreasing [Ca^2+^]_in_ and leading to smooth muscle (or pericyte) relaxation and vasodilation. Dilation of the terminal arteriole (or the initial capillary segment) will result in an increase in blood flow to the microvascular unit, directing blood flow to the site of increased neural activity.

**Figure 4:**
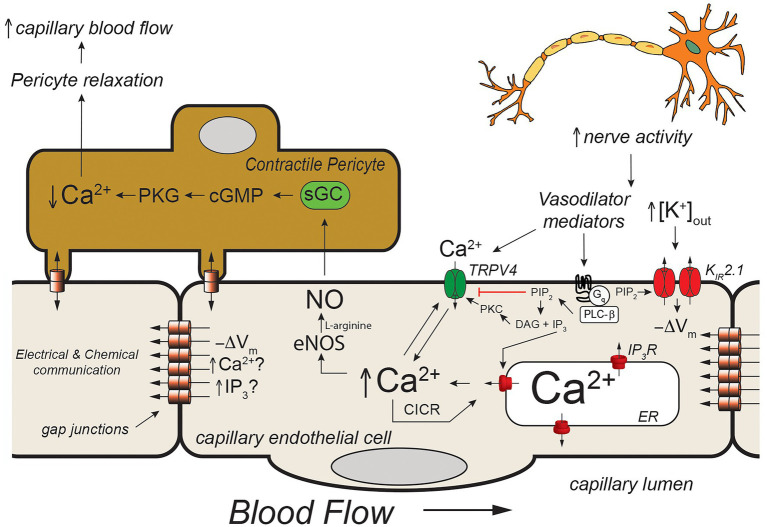
Capillary Ca^2+^ signaling to control pericyte contraction. Schematic of a capillary segment invested with a contractile pericyte. Increased nerve activity will result in accumulation of extracellular K^+^ (as in Figure 2) and a yet unidentified mediator (like PGE_2_, for example). The increase in [K^+^]_out_ will hyperpolarize the membrane (−ΔV_m_) as in Figure 2. The mediator will activate Gα_q_-coupled receptors, leading to hydrolysis of membrane phosphatidylinositol 4,5-bisphosphate (PIP_2_) to form IP_3_ and diacylglycerol (DAG). The IP_3_ will activate IP_3_R in the endoplasmic reticulum resulting in Ca^2+^ release. The increase in [Ca^2+^]_in_, along with reduced membrane PIP_2_, and DAG- and Ca^2+^ activation of protein kinase C (PKC) will activate membrane transient receptor potential vanilloid family member 4 (TRPV4) channels resulting in Ca^2+^ influx which will be bolstered by K_IR_-mediated hyperpolarization. This will further increase local [Ca^2+^]_in_ resulting in additional Ca^2+^ release through IP_3_R (Ca^2+^-induced-Ca^2+^ release; CICR). The resultant increase in [Ca^2+^]_in_ will activate eNOS, increasing production of NO. Nitric oxide will diffuse to the overlying contractile pericyte, activating guanylyl cyclase (GC) to form cGMP, which, in turn, will activate protein kinase G (PKG). Protein kinase G will phosphorylate a number of target proteins resulting decreased [Ca^2+^]_in_ in the pericyte and relaxation. Pericyte relaxation will cause local dilation of the capillary resulting in an increase in blood flow (red cell flux) to this active capillary segment. While the membrane hyperpolarization clearly can be transmitted from endothelial cell-to-endothelial cell as in Figure 2, it is not clear if Ca^2+^ and/or IP_3_ can be transmitted through endothelial cell gap junctions in capillaries to promote cell-to-cell conduction of Ca^2+^ signals. See text for additional information and references.

**Figure 5:**
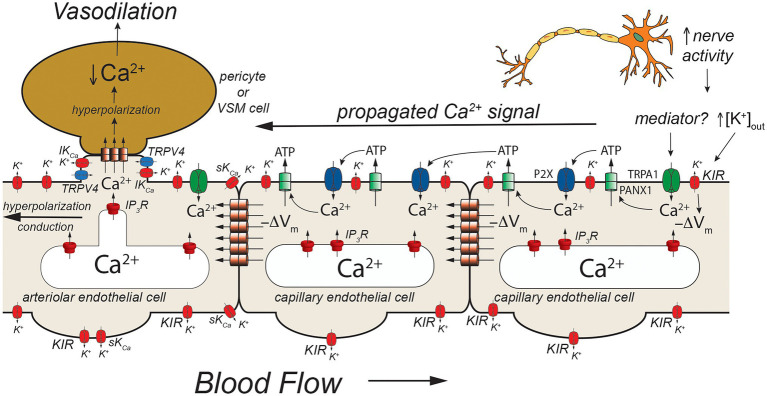
TRPA1- and pannexin-mediated signaling in capillaries. Schematic of a capillary segment that transitions into the initial segment of the capillary coated with contractile pericytes or the terminal arteriole invested with smooth muscle cells. Increased neuron activity leads to increased accumulation of extracellular K^+^ (as in Figures 2, 3) and a yet unidentified mediator. The mediator activates TRPA1 channels in the membrane leading to an increase in [Ca^2+^]_in_ that then activates adjacent pannexin 1 (PANX1) channels which release ATP into the extracellular space. ATP binds to and activates P_2_X receptors resulting in additional Ca^2+^ influx. The additional increase in [Ca^2+^]_in_ activates more PANX1 channels, propagating the Ca^2+^ signal from cell-to-cell. As shown in Figure 2, increased [K^+^]_out_ activates K_IR_ channels causing membrane hyperpolarization (−ΔV_m_). This likely helps to maintain the electrochemical gradient for Ca^2+^ influx through TRPA1 and P_2_X receptors. The hyperpolarization also will be conducted cell-to-cell *via* homocellular gap junctions. When the Ca^2+^ signal reaches endothelial cells in the initial segment of the capillary (coated with contractile pericytes) and also the endothelium in the terminal arteriole, SK_Ca_ and IK_Ca_ channels will be activated resulting in membrane hyperpolarization that is supported by hyperpolarization-induced activation of K_IR_ channels. The hyperpolarization will then be transmitted to overlying contractile mural cells *via* heterocellular gap junctions. In smooth muscle cells (and presumably contractile pericytes), the hyperpolarization will deactivate voltage-gated Ca^2+^ channels, decreasing [Ca^2+^]_in_ and leading to smooth muscle (or pericyte) relaxation and vasodilation. Dilation of the terminal arteriole (or the initial capillary segment) will result in an increase in blood flow to the microvascular unit, directing blood flow to the site of increased neural activity. See text for more information and references.

The functional “unit” of the microcirculation consists of a terminal arteriole, that branches from a network of feed arterioles, and the capillaries perfused by the terminal arteriole (Figure 1). The terminal arteriole is invested with a single layer of contractile vascular smooth muscle cells surrounding an endothelial cell tube (Figure 1). The terminal arteriole transitions and branches into 10–20 capillaries that form the capillary bed (Figure 1). In the cerebral microcirculation, the initial capillary segment consists of an endothelial cell tube coated with contractile pericytes as shown in Figure 1 (Gonzales et al., 2020; Longden et al., 2021; Thakore et al., 2021). Blood flow to the capillaries is controlled both by smooth muscle-induced changes in the diameter of the terminal arterioles (Delashaw and Duling, 1988) and changes in the contractile activity of the pericytes in the initial segment (Longden et al., 2017, 2021; Thakore et al., 2021). Contractile pericytes located at branch points allow fine tuning of blood flow to capillaries adjacent to metabolically active parenchymal cells (neurons and glial cells in the case of the brain microcirculation; Gonzales et al., 2020; Longden et al., 2021). More distal portions of capillaries lose their coat of contractile pericytes and instead are associated with occasional pericytes that are non-contractile (not shown in Figure 1; Gonzales et al., 2020; Longden et al., 2021; Thakore et al., 2021). Capillaries then coalesce into venules that are invested with pericytic smooth muscle cells, rather than the circumferentially oriented arteriolar smooth muscle cells (Jackson, 2012). The remainder of this perspective will focus on endothelial cell ion channels and cell-cell signaling between adjacent endothelial cells and overlying smooth muscle cells in arterioles (Figure 2) and between capillary endothelial cells, arteriolar endothelial cells, and overlying pericytes or smooth muscle cells (Figures 3–5).

## Arteriolar Endothelial Cell Ion Channels and Cell-Cell Communication

Excellent examples of cell-cell communication in the arteriolar wall that depend on endothelial ion channels are endothelium-dependent, agonist-induced vasodilation, myoendothelial feedback, and shear-stress-dependent vasodilation. The primary ion channels involved in these three processes are displayed in Figure 2.

Endothelium-dependent, agonist-induced vasodilation is initiated when vasodilator agonists bind to and activate Gα_q_-coupled receptors, which, in turn, activate phospholipase C β (PLCβ), leading to formation of inositol 1,4,5 trisphosphate (IP_3_) and diacylglycerol (DAG) from membrane phosphatidylinositol 4,5-bisphosphate (PIP_2_) stores (Berridge, 1993; Figure 2). The formed IP_3_ binds to IP_3_ receptors (IP_3_R) in the endoplasmic reticulum membrane, sensitizing them to activation by cytosolic Ca^2+^ to cause Ca^2+^-induced Ca^2+^-release (CICR; Foskett et al., 2007; Mak and Foskett, 2015; Figure 2).

Local subplasmalemmal increases in Ca^2+^ from this process, along with DAG- and Ca^2+^-activated protein kinase C (PKC; Sonkusare et al., 2012) and reduced membrane PIP_2_ levels (Harraz et al., 2018) activate clusters of transient receptor potential vanilloid family member 4 (TRPV4) channels located in myoendothelial projections (MEPs), the site of myoendothelial gap junctions (MEGJs; Earley, 2011; Sonkusare et al., 2012, 2014). In cerebral arterioles, TRPA1 channels also are located in this microdomain (Earley et al., 2009; Qian et al., 2013; Sullivan et al., 2015). Calcium influx through TRPV4 (or TRPA1) channels, visualized as Ca^2+^ sparklets (Sullivan and Earley, 2013), is amplified by CICR through IP_3_R resulting in MEP-localized increases in [Ca^2+^]_in_ termed Ca^2+^ pulsars (Ledoux et al., 2008), which can propagate into larger IP_3_R-mediated Ca^2+^ wavelets (Tran et al., 2012) and even larger whole-cell-based Ca^2+^ waves (Duza and Sarelius, 2004; Kansui et al., 2008; Socha et al., 2012b).

Release of Ca^2+^ through IP_3_R from the endoplasmic reticulum stimulates Ca^2+^ influx through several plasma membrane cation channels that conduct Ca^2+^ (Jackson, 2016). The loss of Ca^2+^ from the endoplasmic reticulum is sensed by stromal interacting molecule 1 (STIM1) resulting in activation of plasma membrane channels composed of ORAI1 channels (Abdullaev et al., 2008; Sundivakkam et al., 2012; Zhou et al., 2014) likely in combination with transient receptor potential C family member 1 (TRPC1; Mehta et al., 2003; Ahmmed et al., 2004; Kwiatek et al., 2006; Sundivakkam et al., 2009, 2012) and/or TRPC4 channels (Freichel et al., 2001; Tiruppathi et al., 2002; Sundivakkam et al., 2012) providing a source of [Ca^2+^]_in_ to help refill endoplasmic reticulum Ca^2+^ stores *via* smooth endoplasmic reticulum Ca^2+^ transporter (SERCA). The agonist-induced DAG production also can activate plasma membrane TRPC3 (Senadheera et al., 2012; Kochukov et al., 2014) and TRPC6 channels (Loga et al., 2013) that can contribute to agonist-induced increases in [Ca^2+^]_in_.

The agonist-induced increases in [Ca^2+^]_in_ mediated by TRPV4 channels (or TRPA1 channels), IP_3_R, and other ion channels activate IK_Ca_ which tend to cluster at MEPs in macromolecular signaling domains (Sonkusare et al., 2012), and SK_Ca_ channels that are more broadly distributed around the periphery of endothelial cells, resulting in K^+^ efflux from the cells and membrane hyperpolarization (Jackson, 2016). Agonist-induced activation of TRPC3 channels may also, in some endothelial cells, specifically activate SK_Ca_ channels that likewise are found in signaling domains in the cell membrane (Kochukov et al., 2014).

The SK_Ca_- and IK_Ca_-initiated hyperpolarization then can be transmitted to overlying smooth muscle cells through MEGJs, deactivating voltage-gated Ca^2+^ channels, resulting in vasodilation (Earley, 2011). Endothelial cell (and vascular smooth muscle cell) inwardly rectifying K^+^ (K_IR_) channels are activated by the SK_Ca_- and IK_Ca_-initiated hyperpolarization, amplifying the hyperpolarization (Sonkusare et al., 2016; Jackson, 2017). The SK_Ca_- and IK_Ca_-initiated hyperpolarization also increases the electrochemical gradient driving Ca^+^ influx through TRPV4 (and all cation channels) another means of amplifying this signaling pathway (Qian et al., 2014).

Because endothelial cells are tightly coupled by homocellular gap junctions (de Wit and Griffith, 2010; Bagher and Segal, 2011), and because of the amplifying effect of K_IR_ channels (Jackson, 2017), endothelial cell hyperpolarization initiated at one end of an arteriole, for example, can be conducted for mm distances along the arteriole which is the electrical basis of the rapid conducted vasodilation that is observed in the microcirculation (de Wit and Griffith, 2010; Bagher and Segal, 2011; Socha et al., 2012a). The arteriolar endothelial cell Ca^2+^ signal also appears to be able to be transmitted from endothelial cell-to-endothelial cell but with a much slower time course (Domeier and Segal, 2007; Uhrenholt et al., 2007; Socha et al., 2012b). The signal (Ca^2+^ and/or IP_3_) that moves through endothelial cell homocellular gap junctions to transmit the Ca^2+^ signal from cell-to-cell has not been established.

In addition to activation of SK_Ca_- and IK_Ca_-initiated hyperpolarization, increased [Ca^2+^]_in_ will activate production and release of endothelial cell autacoids including nitric oxide (NO), prostaglandin I_2_ (prostacyclin; PGI_2_), epoxides of arachidonic acid (EETs), and hydrogen peroxide (H_2_O_2_; Edwards et al., 2010). These can diffuse to overlying smooth muscle cells and induce smooth muscle relaxation and vasodilation through mechanisms that include activation of smooth muscle K^+^ channels (Tykocki et al., 2017). It also should be noted that K^+^-efflux from endothelial cells *via* SK_Ca_ and IK_Ca_ can raise extracellular [K^+^] and activate smooth muscle K_IR_ channels (Edwards et al., 1998) and the Na^+^/K^+^ ATPase (Weston et al., 2002) contributing to endothelium-dependent smooth muscle hyperpolarization and vasodilation in some arteries.

*Ex vivo* studies of isolated resistance arteries and arterioles suggest that the SK_Ca_- and IK_Ca_-initiated endothelial cell hyperpolarization and MEGJ-mediated smooth muscle relaxation and vasodilation are the major form of cell-cell communication during endothelium-dependent vasodilation in these vessels (Edwards et al., 2010). *In vitro* studies of isolated vessels in which endothelial cell and vascular smooth muscle cell membrane potential were measured simultaneously have clearly shown electrical coupling between endothelial cells and smooth muscle cells (Emerson and Segal, 2000, 2001; Yamamoto et al., 2001). However, *in vivo* studies of arteriolar endothelium-dependent vasodilation have suggested that this myoendothelial electrical coupling may not be as prevalent (de Wit et al., 2008; Schmidt and de Wit, 2020). For example, in mouse cremaster muscle arterioles studied by intravital microscopy, acetylcholine-induced hyperpolarization of endothelial cells was inhibited by apamin, but unaffected by iberiotoxin, whereas hyperpolarization of smooth muscle cells was inhibited by apamin (although to a lesser extent than the endothelium) and iberiotoxin (Siegl et al., 2005). If endothelial cells and smooth muscle cells were tightly electrically coupled, apamin should have equally blocked hyperpolarization of both cell types. That iberiotoxin inhibited smooth muscle hyperpolarization suggests that some signal other than hyperpolarization is being transmitted to the smooth muscle cells to activate BK_Ca_ channels. Other studies refute these findings and argue that endothelial cells and vascular smooth muscle cells are well electrically coupled (Wolfle et al., 2011). Additional experiments will be required to resolve these conflicting findings.

Endothelial ion channels and cell-cell communication also are involved in myoendothelial feedback. In arterioles and resistance arteries, increases in smooth muscle tone induced by vasoconstrictors result in an increase in endothelial cell [Ca^2+^]_in_, activation of IK_Ca_ and SK_Ca_ channels to hyperpolarize the endothelial cells, and activation of production of NO and likely other endothelial cell Ca^2+^-dependent vasodilator autacoids [see Lemmey et al. (2020) for a detailed review of this topic]. The hyperpolarization is transmitted back to the smooth muscle through MEGJs, deactivating smooth muscle voltage-gated Ca^2+^ channels, and blunting the vasoconstrictor-induced smooth muscle tone. Endothelium-derived NO (or other vasodilator autacoids) likewise feedback to the smooth muscle limiting vasoconstrictor-induced smooth muscle tone. Studies in mouse mesenteric arteries have shown that vasoconstrictors activate endothelial TRPV4 channels located in MEPs producing TRPV4 Ca^2+^ sparklets that activate MEP-localized IK_Ca_ channels to hyperpolarize the endothelial cells. Calcium release through IP_3_Rs (also located in MEPs) was implicated in the activation of TRPV4 channels during this process (Hong et al., 2018). Endothelial TRPV4 channels in MEPs also have been shown to be activated at low intravascular pressure resulting in TRPV4 Ca^2+^ sparklets, activation of IK_Ca_ channels, and a reduction in myogenic tone (Bagher et al., 2012). The signal that is transmitted from vascular smooth muscle cells to endothelial cells *via* MEGJs to initiate this TRPV4 channel/IP_3_R/IK_Ca_ channel-mediated myoendothelial feedback remains unclear with evidence for both transmission of Ca^2+^ (Garland et al., 2017) and IP_3_ (Tran et al., 2012; Hong et al., 2018) from smooth muscle to endothelial cells to activate MEP-localized TRPV4 channels and/or IP_3_Rs. Additional research will be required to define the signaling molecule responsible for myoendothelial feedback.

Shear-induced dilation of resistance arteries and arterioles also involves cell-cell communication and several endothelial cell ion channels including K_IR_ channels (Ahn et al., 2017; Fancher and Levitan, 2020), TRPV4 channels (Mendoza et al., 2010), PIEZO1 channels (Li et al., 2014; Ranade et al., 2014; Rode et al., 2017), transient receptor potential polycystin family member 1 (TRPP1) channels (MacKay et al., 2020), IP_3_R, SK_Ca_ channels, and IK_Ca_ channels (MacKay et al., 2020; Figure 2).

Barium-induced block of K_IR_ channels or use of K_IR_2.1 heterozygotes were shown to inhibit a significant portion of shear-stress-induced vasodilation in mouse mesenteric arteries that involved phosphorylation of AKT and eNOS and increased NO production, but not activation of SK_Ca_ channels (Ahn et al., 2017). The precise role played by K_IR_2.1 channels in this process remains to be established.

Shear-induced activation of TRPV4 (Mendoza et al., 2010) and/or PIEZO1 (Li et al., 2014; Ranade et al., 2014; Rode et al., 2017) also has been implicated in shear-induced dilation of resistance arteries. It has been postulated that Ca^2+^ influx through these channels increases [Ca^2+^]_in_ to cause both Ca^2+^-dependent production of NO and other endothelial cell vasodilator autacoids and activation of SK_Ca_ and IK_Ca_ channels to produce endothelial cell hyperpolarization and transmission of this hyperpolarization to overlying smooth muscle cells through MEGJs (Fancher and Levitan, 2020).

Shear-induced activation of endothelial cell TRPP1 channels has also been shown to contribute to shear-stress-induced vasodilation (MacKay et al., 2020). Conditional knockout of TRPP1 from endothelial cells was shown to attenuate shear-induced activation of endothelial SK_Ca_ and IK_Ca_ channels as well as smooth muscle hyperpolarization induced by intralumenal flow (MacKay et al., 2020). The eNOS/NO component of shear-stress-induced vasodilation in mouse mesenteric arteries also was reduced by conditional knockout of endothelial cell TRPP1 (MacKay et al., 2020). What remains to be established is how these ion channels (K_IR_2.1, TRPV4, PIEZO1, and TRPP1) integrate into a consolidated view of shear-stress-mediated vasodilation in arterioles and resistance arteries (Fancher and Levitan, 2020).

## Capillary Endothelial Cell Ion Channels and Cell-Cell Communication

Capillaries are the primary site of exchange of respiratory gasses (O_2_ and CO_2_) and substrates (glucose, amino acids, fatty acids, nucleotides, etc.) that support parenchymal cell metabolism (Durán et al., 2011), and recent evidence suggests that these exchange microvessels serve as important sensors of parenchymal cell metabolism which then communicate upstream to segments of the capillary with contractile pericytes and/or to endothelial cells and overlying smooth muscle cells in terminal arterioles to control local vascular resistance such that blood flow is directed to capillaries supplying active parenchymal cells (Murrant and Sarelius, 2015; Longden et al., 2017, 2021; Garcia and Longden, 2020; Zhao et al., 2020). Data from the cerebral microcirculation are particularly compelling. As in arteriolar endothelial cells, cerebral capillary endothelial cells express a diverse array of ion channels involved in their homeostatic functions including: IP_3_R, TRPV4 channels, and K_IR_2.1 channels (Garcia and Longden, 2020; Longden et al., 2021; Figures 3, 4) as well as transient receptor potential ankyrin family member 1 (TRPA1), pannexin 1 (PANX1), and P_2_X purinergic receptors (Thakore et al., 2021; Figure 5). The notable exception is the apparent lack of SK_Ca_ and IK_Ca_ channels from the bulk of capillary endothelial cells (Longden et al., 2017, 2021), although recent evidence suggests that SK_Ca_ and IK_Ca_ channels are expressed in the endothelial cells in the initial segment of capillaries that are covered by contractile pericytes (Thakore et al., 2021).

Using a novel *ex vivo* preparation consisting of a cerebral parenchymal arteriole along with a terminal arteriole and capillaries that arise from these vessels, Longden et al. (2017) showed that application of elevated extracellular K^+^ ([K^+^]_out_ = 10 mM) to the capillaries in this preparation resulted in dilation of the parenchymal arteriole and that this [K^+^]_out_-induced dilation was mediated by endothelial cell K_IR_2.1 channels, confirmed both by sensitivity in Ba^2+^ and also by endothelial cell knockout of K_IR_2.1. They further showed that application of elevated [K^+^]_out_ to cerebral capillaries, *in vivo*, resulted in arteriolar dilation and an increase in capillary red cell flux that could be blocked by Ba^2+^. Importantly, they also demonstrated that functional hyperemia in the whisker barrel cortex (neurovascular coupling) was diminished in endothelial cell K_IR_2.1 knockouts and substantially inhibited by application of Ba^2+^. Together, these data support a model where increases in neural activity result in an elevation in [K^+^]_out_ in the vicinity of the active neurons (Figure 3). The elevated [K^+^]_out_ activates capillary endothelial cell K_IR_2.1 channels, hyperpolarizing the endothelial cells. This hyperpolarization is conducted along the capillary through endothelial cell homocellular gap junctions and facilitated by hyperpolarization-induced activation of upstream K_IR_2.1 channels. Upon reaching the arteriolar endothelium, the K_IR_2.1-mediated hyperpolarization is transmitted to overlying smooth muscle cells *via* MEGJs, hyperpolarizing the smooth muscle cells, and deactivating smooth muscle voltage-gated Ca^2+^ channels, resulting in vasodilation. While elevated [K^+^]_out_ has long been posited as a potential mediator of functional hyperemia (Davis et al., 2011), this was the first study to define exactly where the elevated [K^+^]_out_ is sensed. Whether this model also applies to skeletal muscle where K^+^ also is implicated in functional hyperemia (Armstrong et al., 2007; Crecelius et al., 2014) remains to be established.

*In vivo* studies in mice expressing the genetically encoded Ca^2+^ sensor, GCaMP8 in their endothelial cells has shown that neural activity is associated with endothelial cell [Ca^2+^]_in_ signals that are triggered by neuronal activity and mediated by Ca^2+^ release through IP_3_R. Longden et al. (2021) went on to show that these Ca^2+^ signals could be inhibited by tetrodotoxin (to inhibit neural activity) and reduced by blocking Gα_q_ signaling, K_IR_2.1 channels, and TRPV4 channels. Furthermore, they showed that these capillary Ca^2+^ signals resulted in formation of NO in capillaries covered by contractile pericytes which relaxed the pericytes to direct red cell flux (a surrogate for blood flow) to the capillaries with active Ca^2+^ signals. Their observations suggest a model (Figure 4) in which active neurons release both K^+^ (necessary for repolarization of each nerve action potential) and a small molecule mediator (they suggested PGE_2_) that activates Gα_q_ signaling to produce IP_3_. The Gα_q_ signaling involves PLCβ which hydrolyzes PIP_2_ to form IP_3_ and DAG. This process reduces PIP_2_ levels in the membrane and results in increased TRPV4 channel activity and, at the same time, restrains K_IR_2.1 channel activity (Harraz et al., 2018). The formed IP_3_ sensitizes IP_3_R to CICR which is facilitated by Ca^2+^ entry through the TRPV4 channels. The [K^+^]_out_-induced K_IR_-mediated endothelial cell hyperpolarization enhances the Ca^2+^ influx through TRPV4 channels by increasing the electrochemical gradient for Ca^2+^ influx. The elevated endothelial cell [Ca^2+^]_in_ activates endothelial cell nitric oxide synthase (eNOS), increasing the formation of NO that diffuses to the overlying pericytes, relaxing them to increase capillary diameter and direct blood flow to the actively signaling capillary segment. What remains unclear is how far these Ca^2+^ signals are transmitted along the capillaries and the exact nature of the neuron (or glial cell)-produced “mediator” that activates a capillary Gα_q_ receptor in this scheme. Longden et al. (2021) suggested that the Ca^2+^ events are restricted to single cells and do not conduct between endothelial cells. Given how well endothelial cells are coupled by homocellular gap junctions, this finding is unexpected, because studies in resistance arteries have shown conduction of Ca^2+^ signals between many endothelial cells (Domeier and Segal, 2007; Uhrenholt et al., 2007). Additional research will be required to resolve this issue and determine how distal portions of the capillary which are devoid of contractile mural cells signal to more proximal sections endowed with contractile pericytes if these Ca^2+^ signals indeed do not conduct from cell-to-cell.

The mediator responsible for the Ca^2+^ signals observed by Longden et al. (2021) also remains to be established. They suggested PGE_2_ as a candidate and showed that PGE_2_ could elicit Ca^2+^ signals in capillary endothelial cells. However, subsequent studies using the parenchymal arteriole with attached capillary preparation suggest that PGE_2_ may not be the mediator (Rosehart et al., 2021). In these studies, while PGE_2_ applied to the capillaries elicited signals conducted from the capillaries to the arteriole to produce vasodilation, the authors found that this response was not attenuated by blocking TRPV4 or TRPA1 channels. These data are inconsistent with PGE_2_ being the mediator responsible for the *in vivo* capillary Ca^2+^ signals recorded by Longden et al. (2021) and shown in Figure 4. Additional research will be required to identify the mediator linking neural activity with capillary Ca^2+^ signaling.

An additional model has developed from studies that suggest a role for TRPA1, PANX1, and P_2_X purinergic receptors in cerebral functional hyperemia (Thakore et al., 2021; Figure 5). Thakore and colleagues (Thakore et al., 2021) showed that application of agonists for TRPA1 channels to capillaries in the novel arteriole-capillary preparation noted above, resulted in a slowly conducting signal that could be inhibited by blockers or genetic deletion of PANX1 channels, destruction of extracellular ATP, or block of P_2_X receptors. They went on to show that functional hyperemia in the whisker barrel cortex was inhibited by either a TRPA1 antagonist or genetic knockout of endothelial cell TRPA1 channels. In their model (Figure 5), neural activity produces a mediator that activates TRPA1 channels. The Ca^2+^ influx through TRPA1 channels activates adjacent PANX1 channels, releasing ATP into the extracellular space. The “cloud” of extracellular ATP activates P_2_X receptors increasing intracellular Ca^2+^ and activating additional PANX1 channels producing a slowly propagating Ca^2+^ signal that travels from cell-to-cell to the initial segment of the capillary where SK_Ca_ and IK_Ca_ channels are expressed in the endothelial cells. The increased [Ca^2+^]_in_ activates the SK_Ca_ and IK_Ca_ channels producing a rapidly conducting electrical signal that travels to and through the arteriolar endothelium *via* endothelial cell homocellular gap junctions. This endothelial cell hyperpolarization is then transmitted to overlying smooth muscle cells *via* MEGJs to deactivate voltage-gated Ca^2+^ channels and produce vasodilation. Capillary-to-arteriole signaling in skeletal muscle also has been proposed (Murrant and Sarelius, 2015; Lamb et al., 2021) and may involve pannexins and ATP (Lamb et al., 2021). The exact mediator that triggers activation of TRPA1 channels remains to be found.

In the heart, a novel signaling mechanism involving heterocellular gap junctions between cardiac myocytes and capillary endothelial cells has been proposed to mediate functional hyperemia (Zhao et al., 2020). Zhoa and colleagues propose that increases in cardiac metabolism (increased heart rate and/or contractility) result in activation of cardiac ATP-sensitive K^+^ channels and membrane hyperpolarization. The cardiac myocyte hyperpolarization is transmitted to capillary endothelial cells *via* heterocellular gap junctions. The hyperpolarization is then transmitted along the capillary endothelium *via* homocellular gap junctions to the endothelium in terminal arterioles, which is facilitated by hyperpolarization- and [K^+^]_out_-induced activation of endothelial K_IR_ channels. Once in the arteriolar endothelium, the hyperpolarization is transmitted by MEGJs to overlying smooth muscle cells to cause deactivation of voltage-gated Ca^2+^ channels and vasodilation. Whether other forms of capillary-to-arteriole signaling function in the heart remain to be established.

## Conclusion and Final Perspective

Endothelial cell ion channels importantly contribute to cell-cell communication between cells in the wall of arterioles and between parenchymal cells, capillary endothelial cells, arteriolar endothelial cells, and contractile mural cells (smooth muscle or pericytes). An important question remaining is: how do all of these ion channels fit into a consolidated scheme? For example, are the TRPV4/IP_3_R model (Figure 4) and the TRPA1/PANX1 model (Figure 5) really separate pathways, or are they integrated into a more complicated single scheme of cell-cell communication in the microcirculation? Also, how general are the models presented? Can the pathways identified in the cerebral microcirculation (Figures 3–5) be extended to microvascular beds in other tissues (such as skeletal muscle and the heart) or are separate signaling pathways, such as that described in the heart involving gap junction signaling between cardiac myocytes and capillary endothelial cells, functional? Capillary-to-arteriole signaling has been proposed in skeletal muscle (Murrant and Sarelius, 2015) but critical tests of this hypothesis remain to be performed. While local superfusion experiments have provided evidence for conducted signals and capillary-to-arteriole signaling in skeletal muscle [see Twynstra et al. (2012), for example], interpretation of the findings of such experiments is difficult because skeletal muscle fibers are longer than the scale of microvascular units such that effects of the applied superfusate on skeletal muscle fibers cannot be excluded, for example. Answering the questions outlined above will require additional research and development of new and novel approaches to study cell-cell communication in the microcirculation of organs and tissues around the body.

The effects of disease states on ion channels and cell-cell communication in the microcirculation also is an area that is developing. In the brain, for example, K_IR_ signaling in capillaries (as per Figure 3) is crippled in a murine model of Alzheimer’s disease, a defect that can be improved by application of exogenous PIP_2_ (Mughal et al., 2021). How are the other pathways of capillary-to-arteriole signaling affected in this disease? We already know that capillary TRPV4 channels are activated by PIP_2_ loss (Harraz et al., 2018), does this mean that TRPV4 channel signaling will be increased in cerebral capillaries in Alzheimer’s disease? Is a PIP_2_-related increase in TRPV4 channel activity compensatory to the loss of K_IR_ channel function, or part of the vascular pathology in this neurodegenerative disease? Obviously, additional research will be required to answer these additional questions about endothelial cell ion channels and cell-cell communication in the microcirculation.

## Author Contributions

WJ conceived, wrote, and edited this manuscript. The content is solely the responsibility of the author and does not necessarily represent the official views of the National Institutes of Health.

## Funding

This work was supported by National Heart, Lung and Blood Institute grants HL-137694 and PO1-HL-070687.

## Conflict of Interest

The author declares that the research was conducted in the absence of any commercial or financial relationships that could be construed as a potential conflict of interest.

## Publisher’s Note

All claims expressed in this article are solely those of the authors and do not necessarily represent those of their affiliated organizations, or those of the publisher, the editors and the reviewers. Any product that may be evaluated in this article, or claim that may be made by its manufacturer, is not guaranteed or endorsed by the publisher.

## References

[ref1] Abdullaev I. F. Bisaillon J. M. Potier M. Gonzalez J. C. Motiani R. K. Trebak M. (2008). STIM1 and Orai1 mediate CRAC currents and store-operated calcium entry important for endothelial cell proliferation. Circ. Res. 103, 1289–1299. doi: 10.1161/01.res.0000338496.95579.56, PMID: 18845811 PMC2682347

[ref2] Ahmmed G. U. Mehta D. Vogel S. Holinstat M. Paria B. C. Tiruppathi C.. (2004). Protein kinase calpha phosphorylates the TRPC1 channel and regulates store-operated Ca^2+^ entry in endothelial cells. J. Biol. Chem. 279, 20941–20949. doi: 10.1074/jbc.M313975200, PMID: 15016832

[ref3] Ahn S. J. Fancher I. S. Bian J. T. Zhang C. X. Schwab S. Gaffin R.. (2017). Inwardly rectifying K^+^ channels are major contributors to flow-induced vasodilatation in resistance arteries. J. Physiol. 595, 2339–2364. doi: 10.1113/JP273255, PMID: 27859264 PMC5374117

[ref4] Armstrong M. L. Dua A. K. Murrant C. L. (2007). Potassium initiates vasodilatation induced by a single skeletal muscle contraction in hamster cremaster muscle. J. Physiol. 581, 841–852. doi: 10.1113/jphysiol.2007.130013, PMID: 17363384 PMC2075172

[ref5] Bagher P. Beleznai T. Kansui Y. Mitchell R. Garland C. J. Dora K. A. (2012). Low intravascular pressure activates endothelial cell TRPV4 channels, local Ca^2+^ events, and IKCa channels, reducing arteriolar tone. Proc. Natl. Acad. Sci. U. S. A. 109, 18174–18179. doi: 10.1073/pnas.1211946109, PMID: 23071308 PMC3497745

[ref6] Bagher P. Segal S. S. (2011). Regulation of blood flow in the microcirculation: role of conducted vasodilation. Acta Physiol. 202, 271–284. doi: 10.1111/j.1748-1716.2010.02244.x, PMID: 21199397 PMC3115483

[ref7] Berridge M. (1993). Inositol trisphosphate and calcium signalling. Nature 361, 315–325. doi: 10.1038/361315a0 8381210

[ref8] Crecelius A. R. Luckasen G. J. Larson D. G. Dinenno F. A. (2014). KIR channel activation contributes to onset and steady-state exercise hyperemia in humans. Am. J. Physiol. Heart Circ. Physiol. 307, H782–H791. doi: 10.1152/ajpheart.00212.2014, PMID: 24973385 PMC4187399

[ref9] Davis M. J. Hill M. A. Kuo L. (2011). “Local regulation of microvascular perfusion,” in Comprehensive Physiology. ed. Terjung R. L. (Hoboken, New Jersey: John Wiley & Sons, Inc.)

[ref10] de Wit C. Boettcher M. Schmidt V. J. (2008). Signaling across myoendothelial gap junctions–fact or fiction? Cell Commun. Adhes. 15, 231–245. doi: 10.1080/15419060802440260 18979293

[ref11] de Wit C. Griffith T. M. (2010). Connexins and gap junctions in the EDHF phenomenon and conducted vasomotor responses. Pflugers Arch. 459, 897–914. doi: 10.1007/s00424-010-0830-4, PMID: 20379740

[ref12] Delashaw J. B. Duling B. R. (1988). A study of the functional elements regulating capillary perfusion in striated muscle. Microvasc. Res. 36, 162–171. doi: 10.1016/0026-2862(88)90016-7, PMID: 3185308

[ref13] Domeier T. L. Segal S. S. (2007). Electromechanical and pharmacomechanical signalling pathways for conducted vasodilatation along endothelium of hamster feed arteries. J. Physiol. 579, 175–186. doi: 10.1113/jphysiol.2006.124529, PMID: 17138602 PMC2075370

[ref14] Durán W. N. Sánchez F. A. Breslin J. W. (2011). “Microcirculatory exchange function,” in Comprehensive Physiology. ed. Terjung R. L. (Hoboken, New Jersey: Wiley)

[ref15] Duza T. Sarelius I. H. (2004). Localized transient increases in endothelial cell Ca^2+^ in arterioles in situ: implications for coordination of vascular function. Am. J. Physiol. Heart Circ. Physiol. 286, H2322–H2331. doi: 10.1152/ajpheart.00006.2004, PMID: 14962843

[ref16] Earley S. (2011). Endothelium-dependent cerebral artery dilation mediated by transient receptor potential and Ca^2+^−activated K^+^ channels. J. Cardiovasc. Pharmacol. 57, 148–153. doi: 10.1097/FJC.0b013e3181f580d9, PMID: 20729757

[ref17] Earley S. Gonzales A. L. Crnich R. (2009). Endothelium-dependent cerebral artery dilation mediated by TRPA1 and Ca^2+^-activated K^+^ channels. Circ. Res. 104, 987–994. doi: 10.1161/CIRCRESAHA.108.189530, PMID: 19299646 PMC2966339

[ref18] Edwards G. Dora K. A. Gardener M. J. Garland C. J. Weston A. H. (1998). K^+^ is an endothelium-derived hyperpolarizing factor in rat arteries. Nature 396, 269–272. doi: 10.1038/24388 9834033

[ref19] Edwards G. Feletou M. Weston A. H. (2010). Endothelium-derived hyperpolarising factors and associated pathways: a synopsis. Pflugers Arch. 459, 863–879. doi: 10.1007/s00424-010-0817-1, PMID: 20383718

[ref20] Emerson G. G. Segal S. S. (2000). Electrical coupling between endothelial cells and smooth muscle cells in hamster feed arteries: role in vasomotor control. Circ. Res. 87, 474–479. doi: 10.1161/01.res.87.6.474, PMID: 10988239

[ref21] Emerson G. G. Segal S. S. (2001). Electrical activation of endothelium evokes vasodilation and hyperpolarization along hamster feed arteries. Am. J. Physiol. Heart Circ. Physiol. 280, H160–H167. doi: 10.1152/ajpheart.2001.280.1.H160, PMID: 11123230

[ref22] Fancher I. S. Levitan I. (2020). Endothelial inwardly-rectifying K^+^ channels as a key component of shear stress-induced mechanotransduction. Curr. Top. Membr. 85, 59–88. doi: 10.1016/bs.ctm.2020.02.002, PMID: 32402645

[ref23] Foskett J. K. White C. Cheung K. H. Mak D. O. (2007). Inositol trisphosphate receptor Ca^2+^ release channels. Physiol. Rev. 87, 593–658. doi: 10.1152/physrev.00035.2006, PMID: 17429043 PMC2901638

[ref24] Freichel M. Suh S. H. Pfeifer A. Schweig U. Trost C. Weissgerber P.. (2001). Lack of an endothelial store-operated Ca^2+^ current impairs agonist-dependent vasorelaxation in TRP4^−/−^ mice. Nat. Cell Biol. 3, 121–127. doi: 10.1038/35055019, PMID: 11175743

[ref25] Garcia D. C. G. Longden T. A. (2020). Ion channels in capillary endothelium. Curr. Top. Membr. 85, 261–300. doi: 10.1016/bs.ctm.2020.01.005, PMID: 32402642

[ref26] Garland C. J. Bagher P. Powell C. Ye X. Lemmey H. A. L. Borysova L.. (2017). Voltage-dependent Ca^2+^ entry into smooth muscle during contraction promotes endothelium-mediated feedback vasodilation in arterioles. Sci. Signal. 10:eaal3806. doi: 10.1126/scisignal.aal3806, PMID: 28676489

[ref27] Gonzales A. L. Klug N. R. Moshkforoush A. Lee J. C. Lee F. K. Shui B.. (2020). Contractile pericytes determine the direction of blood flow at capillary junctions. Proc. Natl. Acad. Sci. U. S. A. 117, 27022–27033. doi: 10.1073/pnas.1922755117, PMID: 33051294 PMC7604512

[ref28] Harraz O. F. Longden T. A. Hill-Eubanks D. Nelson M. T. (2018). PIP2 depletion promotes TRPV4 channel activity in mouse brain capillary endothelial cells. eLife 7:e38689. doi: 10.7554/eLife.38689, PMID: 30084828 PMC6117155

[ref29] Hong K. Cope E. L. DeLalio L. J. Marziano C. Isakson B. E. Sonkusare S. K. (2018). TRPV4 (transient receptor potential vanilloid 4) channel-dependent negative feedback mechanism regulates Gq protein-coupled receptor-induced vasoconstriction. Arterioscler. Thromb. Vasc. Biol. 38, 542–554. doi: 10.1161/ATVBAHA.117.310038, PMID: 29301784 PMC5823749

[ref30] Jackson W. F. (2012). “Microcirculation,” in Muscle. eds. Olson J. A. Hill E. N. (Boston/Waltham: Academic Press), 1197–1206.

[ref31] Jackson W. F. (2016). “Endothelial cell ion channel expression and function in arterioles and resistance arteries,” in Vascular Ion Channels in Physiology and Disease. eds. Levitan I. Dopico A. M. (Switzerland: Springer International Publishing), 3–36.

[ref32] Jackson W. F. (2017). Boosting the signal: endothelial inward rectifier K^+^ channels. Microcirculation 24:e12319. doi: 10.1111/micc.12319, PMID: 27652592 PMC5360557

[ref33] Kansui Y. Garland C. J. Dora K. A. (2008). Enhanced spontaneous Ca^2+^ events in endothelial cells reflect signalling through myoendothelial gap junctions in pressurized mesenteric arteries. Cell Calcium 44, 135–146. doi: 10.1016/j.ceca.2007.11.012, PMID: 18191200 PMC2635531

[ref34] Kochukov M. Y. Balasubramanian A. Abramowitz J. Birnbaumer L. Marrelli S. P. (2014). Activation of endothelial transient receptor potential C3 channel is required for small conductance calcium-activated potassium channel activation and sustained endothelial hyperpolarization and vasodilation of cerebral artery. J. Am. Heart Assoc. 3:e00091. doi: 10.1161/JAHA.114.000913, PMID: 25142058 PMC4310376

[ref35] Kwiatek A. M. Minshall R. D. Cool D. R. Skidgel R. A. Malik A. B. Tiruppathi C. (2006). Caveolin-1 regulates store-operated Ca^2+^ influx by binding of its scaffolding domain to transient receptor potential channel-1 in endothelial cells. Mol. Pharmacol. 70, 1174–1183. doi: 10.1124/mol.105.021741, PMID: 16822931

[ref36] Lamb I. R. Novielli-Kuntz N. M. Murrant C. L. (2021). Capillaries communicate with the arteriolar microvascular network by a pannexin/purinergic-dependent pathway in hamster skeletal muscle. Am. J. Physiol. Heart Circ. Physiol. 320, H1699–H1711. doi: 10.1152/ajpheart.00493.2020, PMID: 33606585

[ref37] Laughlin M. H. Davis M. J. Secher N. H. van Lieshout J. J. Arce-Esquivel A. A. Simmons G. H.. (2011). Peripheral circulation. Compr. Physiol. 2, 321–447. doi: 10.1002/cphy.c100048 23728977

[ref38] Ledoux J. Taylor M. S. Bonev A. D. Hannah R. M. Solodushko V. Shui B.. (2008). Functional architecture of inositol 1,4,5-trisphosphate signaling in restricted spaces of myoendothelial projections. Proc. Natl. Acad. Sci. U. S. A. 105, 9627–9632. doi: 10.1073/pnas.0801963105, PMID: 18621682 PMC2474537

[ref39] Lemmey H. A. L. Garland C. J. Dora K. A. (2020). Intrinsic regulation of microvascular tone by myoendothelial feedback circuits. Curr. Top. Membr. 85, 327–355. doi: 10.1016/bs.ctm.2020.01.004, PMID: 32402644

[ref40] Li J. Hou B. Tumova S. Muraki K. Bruns A. Ludlow M. J.. (2014). Piezo1 integration of vascular architecture with physiological force. Nature 515, 279–282. doi: 10.1038/nature13701, PMID: 25119035 PMC4230887

[ref41] Loga F. Domes K. Freichel M. Flockerzi V. Dietrich A. Birnbaumer L.. (2013). The role of cGMP/cGKI signalling and Trpc channels in regulation of vascular tone. Cardiovasc. Res. 100, 280–287. doi: 10.1093/cvr/cvt176, PMID: 23832809 PMC3797626

[ref42] Longden T. A. Dabertrand F. Koide M. Gonzales A. L. Tykocki N. R. Brayden J. E.. (2017). Capillary K^+^-sensing initiates retrograde hyperpolarization to increase local cerebral blood flow. Nat. Neurosci. 20, 717–726. doi: 10.1038/nn.4533, PMID: 28319610 PMC5404963

[ref43] Longden T. A. Mughal A. Hennig G. W. Harraz O. F. Shui B. Lee F. K.. (2021). Local IP3 receptor-mediated Ca^2+^ signals compound to direct blood flow in brain capillaries. Sci. Adv. 7:eabh0101. doi: 10.1126/sciadv.abh0101, PMID: 34290098 PMC8294755

[ref44] MacKay C. E. Leo M. D. Fernandez-Pena C. Hasan R. Yin W. Mata-Daboin A.. (2020). Intravascular flow stimulates PKD2 (polycystin-2) channels in endothelial cells to reduce blood pressure. eLife 9:e56655. doi: 10.7554/eLife.56655, PMID: 32364494 PMC7228764

[ref45] Mak D. O. Foskett J. K. (2015). Inositol 1,4,5-trisphosphate receptors in the endoplasmic reticulum: a single-channel point of view. Cell Calcium 58, 67–78. doi: 10.1016/j.ceca.2014.12.008, PMID: 25555684 PMC4458407

[ref46] Mehta D. Ahmmed G. U. Paria B. C. Holinstat M. Voyno-Yasenetskaya T. Tiruppathi C.. (2003). RhoA interaction with inositol 1,4,5-trisphosphate receptor and transient receptor potential channel-1 regulates Ca^2+^ entry. Role in signaling increased endothelial permeability. J. Biol. Chem. 278, 33492–33500. doi: 10.1074/jbc.M302401200, PMID: 12766172

[ref47] Mendoza S. A. Fang J. Gutterman D. D. Wilcox D. A. Bubolz A. H. Li R.. (2010). TRPV4-mediated endothelial Ca^2+^ influx and vasodilation in response to shear stress. Am. J. Physiol. Heart Circ. Physiol. 298, H466–H476. doi: 10.1152/ajpheart.00854.2009, PMID: 19966050 PMC2822567

[ref48] Molica F. Figueroa X. F. Kwak B. R. Isakson B. E. Gibbins J. M. (2018). Connexins and pannexins in vascular function and disease. Int. J. Mol. Sci. 19:1663. doi: 10.3390/ijms19061663, PMID: 29874791 PMC6032213

[ref49] Mughal A. Harraz O. F. Gonzales A. L. Hill-Eubanks D. Nelson M. T. (2021). PIP2 improves cerebral blood flow in a mouse model of Alzheimer’s disease. Function 2:zqab010. doi: 10.1093/function/zqab010, PMID: 33763649 PMC7955025

[ref50] Murrant C. L. Sarelius I. H. (2015). Local control of blood flow during active hyperaemia: what kinds of integration are important? J. Physiol. 593, 4699–4711. doi: 10.1113/JP270205, PMID: 26314391 PMC4626542

[ref51] Nilius B. Droogmans G. (2001). Ion channels and their functional role in vascular endothelium. Physiol. Rev. 81, 1415–1459. doi: 10.1152/physrev.2001.81.4.1415 11581493

[ref52] Qian X. Francis M. Kohler R. Solodushko V. Lin M. Taylor M. S. (2014). Positive feedback regulation of agonist-stimulated endothelial Ca^2+^ dynamics by KCa3.1 channels in mouse mesenteric arteries. Arterioscler. Thromb. Vasc. Biol. 34, 127–135. doi: 10.1161/ATVBAHA.113.302506, PMID: 24177326 PMC4181598

[ref53] Qian X. Francis M. Solodushko V. Earley S. Taylor M. S. (2013). Recruitment of dynamic endothelial Ca^2+^ signals by the TRPA1 channel activator AITC in rat cerebral arteries. Microcirculation 20, 138–148. doi: 10.1111/micc.12004, PMID: 22928941 PMC3524345

[ref54] Ranade S. S. Qiu Z. Woo S. H. Hur S. S. Murthy S. E. Cahalan S. M.. (2014). Piezo1, a mechanically activated ion channel, is required for vascular development in mice. Proc. Natl. Acad. Sci. U. S. A. 111, 10347–10352. doi: 10.1073/pnas.1409233111, PMID: 24958852 PMC4104881

[ref55] Rode B. Shi J. Endesh N. Drinkhill M. J. Webster P. J. Lotteau S. J.. (2017). Piezo1 channels sense whole body physical activity to reset cardiovascular homeostasis and enhance performance. Nat. Commun. 8:350. doi: 10.1038/s41467-017-00429-3, PMID: 28839146 PMC5571199

[ref56] Rosehart A. C. Longden T. A. Weir N. Fontaine J. T. Joutel A. Dabertrand F. (2021). Prostaglandin E2 dilates intracerebral arterioles when applied to capillaries: implications for small vessel diseases. Front. Aging Neurosci. 13:695965. doi: 10.3389/fnagi.2021.695965, PMID: 34483880 PMC8414797

[ref57] Schmidt K. de Wit C. (2020). Endothelium-derived hyperpolarizing factor and myoendothelial coupling: the *in vivo* perspective. Front. Physiol. 11:602930. doi: 10.3389/fphys.2020.602930, PMID: 33424626 PMC7786115

[ref58] Senadheera S. Kim Y. Grayson T. H. Toemoe S. Kochukov M. Y. Abramowitz J.. (2012). Transient receptor potential canonical type 3 channels facilitate endothelium-derived hyperpolarization-mediated resistance artery vasodilator activity. Cardiovasc. Res. 95, 439–447. doi: 10.1093/cvr/cvs208, PMID: 22721989 PMC3422079

[ref59] Siegl D. Koeppen M. Wolfle S. E. Pohl U. de Wit C. (2005). Myoendothelial coupling is not prominent in arterioles within the mouse cremaster microcirculation *in vivo*. Circ. Res. 97, 781–788. doi: 10.1161/01.RES.0000186193.22438.6c 16166558

[ref60] Socha M. J. Behringer E. J. Segal S. S. (2012a). Calcium and electrical signalling along endothelium of the resistance vasculature. Basic Clin. Pharmacol. Toxicol. 110, 80–86. doi: 10.1111/j.1742-7843.2011.00798.x, PMID: 21917120 PMC3271116

[ref61] Socha M. J. Domeier T. L. Behringer E. J. Segal S. S. (2012b). Coordination of intercellular Ca^2+^ signaling in endothelial cell tubes of mouse resistance arteries. Microcirculation 19, 757–770. doi: 10.1111/micc.12000, PMID: 22860994 PMC3502682

[ref62] Sonkusare S. K. Bonev A. D. Ledoux J. Liedtke W. Kotlikoff M. I. Heppner T. J.. (2012). Elementary Ca^2+^ signals through endothelial TRPV4 channels regulate vascular function. Science 336, 597–601. doi: 10.1126/science.1216283, PMID: 22556255 PMC3715993

[ref63] Sonkusare S. K. Dalsgaard T. Bonev A. D. Hill-Eubanks D. C. Kotlikoff M. I. Scott J. D.. (2014). AKAP150-dependent cooperative TRPV4 channel gating is central to endothelium-dependent vasodilation and is disrupted in hypertension. Sci. Signal. 7:ra66. doi: 10.1126/scisignal.2005052, PMID: 25005230 PMC4403000

[ref64] Sonkusare S. K. Dalsgaard T. Bonev A. D. Nelson M. T. (2016). Inward rectifier potassium (Kir2.1) channels as end-stage boosters of endothelium-dependent vasodilators. J. Physiol. 594, 3271–3285. doi: 10.1113/JP271652, PMID: 26840527 PMC4908010

[ref65] Sullivan M. N. Earley S. (2013). TRP channel Ca^2+^ sparklets: fundamental signals underlying endothelium-dependent hyperpolarization. Am. J. Phys. Cell Phys. 305, C999–C1008. doi: 10.1152/ajpcell.00273.2013, PMID: 24025865 PMC3840200

[ref66] Sullivan M. N. Gonzales A. L. Pires P. W. Bruhl A. Leo M. D. Li W.. (2015). Localized TRPA1 channel Ca^2+^ signals stimulated by reactive oxygen species promote cerebral artery dilation. Sci. Signal. 8:ra2. doi: 10.1126/scisignal.2005659, PMID: 25564678 PMC4745898

[ref67] Sundivakkam P. C. Freichel M. Singh V. Yuan J. P. Vogel S. M. Flockerzi V.. (2012). The Ca^2+^ sensor stromal interaction molecule 1 (STIM1) is necessary and sufficient for the store-operated Ca^2+^ entry function of transient receptor potential canonical (TRPC) 1 and 4 channels in endothelial cells. Mol. Pharmacol. 81, 510–526. doi: 10.1124/mol.111.074658, PMID: 22210847 PMC3310414

[ref68] Sundivakkam P. C. Kwiatek A. M. Sharma T. T. Minshall R. D. Malik A. B. Tiruppathi C. (2009). Caveolin-1 scaffold domain interacts with TRPC1 and IP3R3 to regulate Ca^2+^ store release-induced Ca^2+^ entry in endothelial cells. Am. J. Phys. Cell Phys. 296, C403–C413. doi: 10.1152/ajpcell.00470.2008, PMID: 19052258 PMC2660268

[ref69] Thakore P. Alvarado M. G. Ali S. Mughal A. Pires P. W. Yamasaki E.. (2021). Brain endothelial cell TRPA1 channels initiate neurovascular coupling. eLife 10:e63040. doi: 10.7554/eLife.63040, PMID: 33635784 PMC7935492

[ref70] Tiruppathi C. Freichel M. Vogel S. M. Paria B. C. Mehta D. Flockerzi V.. (2002). Impairment of store-operated Ca^2+^ entry in TRPC4^−/−^ mice interferes with increase in lung microvascular permeability. Circ. Res. 91, 70–76. doi: 10.1161/01.RES.0000023391.40106.A8, PMID: 12114324

[ref71] Tran C. H. Taylor M. S. Plane F. Nagaraja S. Tsoukias N. M. Solodushko V.. (2012). Endothelial Ca^2+^ wavelets and the induction of myoendothelial feedback. Am. J. Phys. Cell Phys. 302, C1226–C1242. doi: 10.1152/ajpcell.00418.2011, PMID: 22277756 PMC3330726

[ref72] Twynstra J. Ruiz D. A. Murrant C. L. (2012). Functional coordination of the spread of vasodilations through skeletal muscle microvasculature: implications for blood flow control. Acta Physiol. 206, 229–241. doi: 10.1111/j.1748-1716.2012.02465.x, PMID: 22726936

[ref73] Tykocki N. R. Boerman E. M. Jackson W. F. (2017). Smooth muscle ion channels and regulation of vascular tone in resistance arteries and arterioles. Compr. Physiol. 7, 485–581. doi: 10.1002/cphy.c160011, PMID: 28333380 PMC5575875

[ref74] Uhrenholt T. R. Domeier T. L. Segal S. S. (2007). Propagation of calcium waves along endothelium of hamster feed arteries. Am. J. Physiol. Heart Circ. Physiol. 292, H1634–H1640. doi: 10.1152/ajpheart.00605.2006, PMID: 17098832

[ref75] Weston A. H. Richards G. R. Burnham M. P. Feletou M. Vanhoutte P. M. Edwards G. (2002). K^+^-induced hyperpolarization in rat mesenteric artery: identification, localization and role of Na^+^/K^+^-ATPases. Br. J. Pharmacol. 136, 918–926. doi: 10.1038/sj.bjp.0704787, PMID: 12110616 PMC1573416

[ref76] Wolfle S. E. Chaston D. J. Goto K. Sandow S. L. Edwards F. R. Hill C. E. (2011). Non-linear relationship between hyperpolarisation and relaxation enables long distance propagation of vasodilatation. J. Physiol. 589, 2607–2623. doi: 10.1113/jphysiol.2010.202580, PMID: 21486765 PMC3115829

[ref77] Yamamoto Y. Klemm M. F. Edwards F. R. Suzuki H. (2001). Intercellular electrical communication among smooth muscle and endothelial cells in Guinea-pig mesenteric arterioles. J. Physiol. 535, 181–195. doi: 10.1111/j.1469-7793.2001.00181.x, PMID: 11507168 PMC2278769

[ref78] Zhao G. Joca H. C. Nelson M. T. Lederer W. J. (2020). ATP- and voltage-dependent electro-metabolic signaling regulates blood flow in heart. Proc. Natl. Acad. Sci. U. S. A. 117, 7461–7470. doi: 10.1073/pnas.1922095117, PMID: 32170008 PMC7132126

[ref79] Zhou M.-H. Zheng H. Si H. Jin Y. Peng J. M. He L.. (2014). Stromal interaction molecule 1 (STIM1) and Orai1 mediate histamine-evoked calcium entry and nuclear factor of activated T-cells (NFAT) signaling in human umbilical vein endothelial cells. J. Biol. Chem. 289, 29446–29456. doi: 10.1074/jbc.M114.578492, PMID: 25190815 PMC4200292

